# Therapeutic Targets for Hepatic Fibrosis Driven by Hypoxia-Mediated Hepatic Stellate Cell Activation

**DOI:** 10.3390/biom16081089

**Published:** 2026-07-25

**Authors:** Wenteng Li, Tong Li, Jingwei Mao

**Affiliations:** Department of Gastroenterology, The First Affiliated Hospital of Dalian Medical University, Dalian 116011, China; liwt01@dmu.edu.cn (W.L.); litong2024@dmu.edu.cn (T.L.)

**Keywords:** low oxygen, hypoxia-inducible factor, hepatic stellate cells, liver fibrosis, therapeutic target

## Abstract

Hepatic fibrosis is a pathological process involving the systemic reconfiguration of the liver microenvironment under chronic liver injury. It is a pathological wound healing response characterized by the excessive deposition of extracellular matrix (ECM) driven by the activation of hepatic stellate cells (HSCs), starting with the capillarization of liver sinusoidal endothelial cells (LSECs). This process can lead to liver structure destruction, portal hypertension, and even liver dysfunction, and is a major driving factor for death related to liver diseases. During this process, hypoxia initiates the transcriptional upregulation of effector molecules such as vascular endothelial growth factor (VEGF) and Lysyl oxidase (LOX) by stabilizing hypoxia-inducible factors (HIFs), inducing changes in LSEC phenotype and activation of HSCs, thus becoming a core driving factor. Activated HSCs not only exacerbate the capillarization of LSECs and tissue hypoxia but also strengthen the transcriptional activity of HIFs through autocrine/paracrine signaling, establishing a positive feedback loop linking hypoxia to HIFs and HSC activation, continuously driving the progression of liver fibrosis and significantly increasing the probability of chronic liver diseases evolving into cirrhosis and the risk of death. This review will analyze the mechanism of liver fibrosis driven by hypoxia, integrate the current treatment landscape, focus on exploring the therapeutic targets related to hypoxia-induced activation of hepatic stellate cells leading to liver fibrosis, and evaluate their translational potential. It is expected to provide information for future effective anti-fibrotic intervention strategies.

## 1. Introduction

Hepatic fibrosis represents a reversible wound-healing response to persistent liver cell damage induced by diverse etiologies. Its distinguishing characteristic is the abnormal accumulation of ECM [1,2]. In a normal liver, the synthesis and degradation of ECM uphold a dynamic equilibrium. Fibrosis subsequent to acute injury is reversible; however, chronic injury causes the synthesis of ECM to surpass its degradation, leading to the thickening of fibrous septa and an increase in collagen cross-linking. This process ultimately results in the formation of hepatic fibrosis and may advance to liver cirrhosis, portal hypertension, and liver failure, thereby significantly elevating the risk of hepatocellular carcinoma (HCC) [3]. The factors leading to persistent hepatocyte damage are diverse, mainly including chronic viral hepatitis (HBV, HCV), long-term alcohol abuse, metabolic dysfunction-associated fatty liver disease (MASLD, formerly known as NAFLD), cholestatic diseases, autoimmune diseases, and drug- or toxin-induced injury. In addition, as the prevalence of metabolic syndrome-related metabolic dysfunction-associated fatty liver disease (MAFLD)/nonalcoholic steatohepatitis (NASH) continues to rise, the resulting liver fibrosis has become a significant global health burden [4]. In 2023, the multi-society Delphi consensus renamed NAFLD to MASLD, emphasizing the central role of metabolic dysfunction [5]. The current global prevalence of MASLD is 38% and is projected to rise to 55.4% by 2040, with the highest regional rates observed in Latin America (44.4%) and the Middle East/North Africa (MENA) region (39.4%) [6]. In China, the prevalence of MASLD and MASH, as well as the annual incidence of MASLD, also remain high, at 30.4%, 6.7%, and 37 cases per 1000 person-years, respectively [7]. In addition to the prevalence of MASLD, a 2025 meta-analysis revealed an even more alarming finding: MASLD increased the risk of HCC by 2.34-fold in the general population and by 1.36-fold in patients with chronic hepatitis B [8]. Currently, the only cure for end-stage liver disease is liver transplantation. In the United States, NASH/MASH has become the leading reason for liver transplantation in women (2016) and HCC patients (31% in 2022), but severe organ supply shortages and surgical risks limit treatment [9,10]. This suggests that we need to strengthen the management of metabolic diseases to reduce the demand for liver transplantation in the future, and on this basis, it is necessary and urgent to explore the mechanism of the occurrence and development of liver fibrosis and formulate effective targeted therapy.

Current studies have confirmed that the core event of liver fibrosis is the activation of HSCs. In a healthy liver, resting HSCs are the main reservoir of vitamin A and are involved in the regulation of sinusoid blood flow. When liver cells are injured, hypoxia activates the upregulation of hypoxia-inducible factor-1α (HIF-1α), which capillarizes liver sinusoidal endothelial cells, and then directly differentiates or “activates” HSCs into proliferative, fibroblastic myofibroblasts through a series of reactions. Activated HSCs continuously promote ECM accumulation, microcirculation disturbance and tissue hypoxia through multiple pathogenic mechanisms, such as ECM deposition, hepatic sinusoidal contraction, pro-angiogenesis and positive feedback signal amplification, which eventually promote liver fibrosis to a pathological outcome that is difficult to reverse [11,12,13].

As the core cellular engine driving the progression of liver fibrosis, HSCs are prime targets for anti-fibrosis therapy. However, due to the highly redundant regulatory signal network of HSCs, single-pathway inhibition can readily induce compensatory activation. Simultaneously, the targeted molecules of HSCs are widely expressed, and systemic inhibition may result in off-target toxicity. Moreover, the heterogeneity of HSCs and the dynamic characteristics of liver fibrosis pose significant challenges in the development of universal anti-fibrosis therapies [14,15,16]. Therefore, despite existing strategies focusing on inhibiting HSC activation, inducing their apoptosis, or promoting their reversion, direct anti-fibrotic drugs targeting HSCs have yet to be approved [17].

It is difficult to break through the bottleneck of efficacy by targeting HSCs alone, which suggests starting from the upstream microenvironment to find a new intervention node for the treatment of liver fibrosis. For example, tissue hypoxia is a common feature of the fibrotic microenvironment. Hypoxia-induced stabilization of HIFs drives the activation of HSCs, ultimately leading to exacerbated hypoxia and thereby forming a self-perpetuating positive feedback loop that exacerbates liver fibrosis. Targeting tissue hypoxia may provide an effective strategy to solve this dilemma [18,19].

## 2. Hypoxia and Liver Microenvironment in Fibrosis

**Figure 1:**
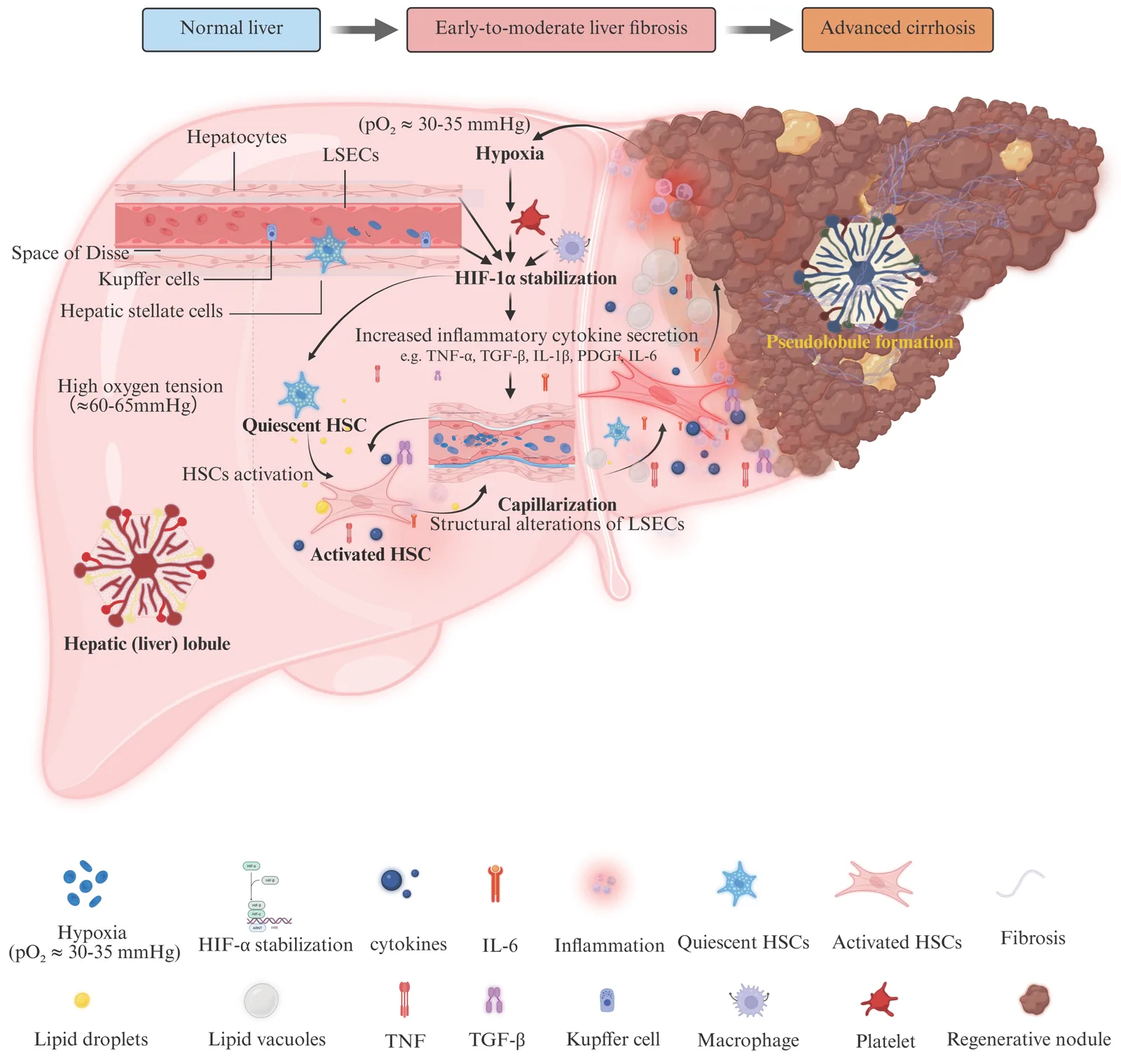
Hypoxia-driven activation of HSCs during liver fibrogenesis. Under physiological conditions, the hepatic sinusoidal microenvironment is relatively oxygenated, with intact LSECs, fenestrae, and an undisturbed Space of Disse, thereby maintaining HSCs in a quiescent, vitamin A-storing state. During chronic liver injury, local hypoxia develops and stabilizes HIF-1α, which promotes the release of pro-inflammatory and pro-fibrogenic mediators, including TNF-α, TGF-β, IL-1β, PDGF, and IL-6, from hepatocytes, Kupffer cells, platelets, and other inflammatory cells. In parallel, hypoxia induces structural alterations in LSECs, including capillarization, loss of fenestrae, basement membrane deposition, and reduced nitric oxide production, which further aggravate sinusoidal dysfunction and microcirculatory impairment. These changes drive the transdifferentiation of quiescent HSCs into activated myofibroblast-like cells characterized by α-SMA expression and enhanced production of extracellular matrix components, such as collagen types I and III, TIMP-1, and additional profibrogenic cytokines. Persistent HSC activation leads to perisinusoidal fibrosis, progressive architectural remodeling, regenerative nodule formation, portal hypertension, and ultimately advanced cirrhosis and liver failure. Arrows in the figure indicate positive regulation or activation of the depicted processes. Created in BioRender. LI, W. (2026). (For computer program and AI disclosure, see the statement preceding the figures.)

In the normal liver, a physiological oxygen gradient along the hepatic acinus establishes and maintains metabolic zonation [20,21]. This gradient supports region-specific functions, and the moderate activation of HIF-2α (a member of the hypoxia-inducible factor family, whose structure and oxygen-sensing mechanism are detailed in Section 3) in the physiological hypoxic zone is important for coordinating hepatocyte proliferation and vascular adaptation during liver regeneration. However, when liver fibrosis disrupts the hepatic oxygenation process, oxygen homeostasis is severely disturbed [22]. Hypoxia then becomes not merely a consequence of fibrosis but an active driver that promotes disease progression [23,24]. The hypoxia-mediated cascade driving hepatic stellate cell (HSC) activation during liver fibrogenesis is illustrated in Figure 1.

However, the occurrence of liver fibrosis disrupts the liver oxygenation process, thereby destroying this delicate oxygen balance. As a result, the stability of HIFs, the core oxygen-sensing transcription factors, is disrupted, leading to the transcriptional activation of downstream target genes that amplify the pathological hypoxia signal and further damage the liver structure [25]. Studies have demonstrated that hypoxia directly induces the activation of HSCs via the HIF signaling pathway. It also stimulates hypoxic hepatocytes to secrete CXCL12, PDGF, PAI-1, etc., forming a pro-fibrotic signaling network and simultaneously inducing epithelial–mesenchymal transition (EMT), thus synergistically driving fibrosis [26,27]. Activated HSCs further damage the liver structure and synergistically exacerbate tissue hypoxia through various means. Notably, in addition to enhancing the transcriptional activity of HIFs through positive feedback from paracrine signals (e.g., TGF-β1 and ROS), activated HSCs can also drive their own activation and ECM production. A vicious cycle is thus formed: liver structural disruption leads to hypoxia, which leads to increased HIF activity and promotes the activation of HSCs, leading to more severe structural disruption and increased hypoxia [26,27]. Once this vicious cycle is initiated, the fibrotic process continues even after the initial injury is addressed. The hypoxic microenvironment is not only a consequence of liver fibrosis, but also a catalyst for its persistent progression. Therefore, targeting the hypoxic response—especially the core factor of HIFs—may serve as a key therapeutic approach to prevent or even reverse liver fibrosis.

## 3. HIFs: Molecular Mediators of Oxygen Sensing

The hypoxia-inducible factor (HIF) family serves as the core oxygen-sensing transcriptional system in mammalian cells. HIFs are heterodimeric transcription factors composed of an oxygen-labile α subunit (HIF-1α, HIF-2α, or HIF-3α) and a constitutively expressed β subunit (aryl hydrocarbon receptor nuclear translocator, ARNT) [28]. Under normoxic conditions, HIF-α is hydroxylated by prolyl hydroxylase domain-containing proteins (PHDs), which utilize O_2_ as a co-substrate. This hydroxylation creates a recognition site for the von Hippel-Lindau (VHL) E3 ubiquitin ligase complex, leading to ubiquitination and subsequent proteasomal degradation [29,30]. Under hypoxia, inactivated PHDs lead to reduced degradation of the HIF-α subunit, which accumulates and translocates to the cell nucleus to dimerize with HIF-β. The heterodimer then binds to hypoxia-response elements (HREs) in the promoter regions of target genes, initiating widespread transcriptional responses that regulate processes such as glycolysis, angiogenesis, erythropoiesis, and activation of hepatic stellate cells [28].

In the context of liver fibrosis, HIFs regulate the expression of downstream molecules such as C-X-C chemokine receptor type 4 (CXCR4) and C-X-C chemokine receptor type 7 (CXCR7), which promote capillarization of LSECs [1,26]. LSEC capillarization impedes oxygen exchange between sinusoidal blood and the hepatocyte parenchyma. In parallel, the formation of dense fibrotic septa in late-stage fibrosis compresses sinusoids and increases the oxygen diffusion distance. Moreover, aberrant arteriovenous shunts, driven by portal hypertension and pathological angiogenesis, bypass the parenchymal microcirculation. Together, these changes aggravate tissue ischemia and hypoxia. Simultaneously, HIFs upregulate their downstream target genes, including chemokine (C-C motif) receptor 1 gene (*Ccr1*), interleukin-13 receptor subunit alpha-1 (*Il13ra1*), *VEGF*, and prolyl 4-hydroxylase subunit alpha-2 (*P4ha2*), which encode chemokine receptors, pro-angiogenic factors, and collagen-synthesizing enzymes. HIFs also induce the expression of pro-fibrotic cytokines such as TGF-β1, CTGF, and interleukin-6 (IL-6). Through a feed-forward loop, this further promotes the release of additional pro-fibrotic factors, including PDGF-BB and TGF-β itself. Collectively, these events drive and accelerate HSC activation and ECM deposition, thereby propelling the fibrotic process and ultimately exacerbating local tissue hypoxia [12].

Notably, HIF-1α and HIF-2α—the two most extensively studied isoforms—are not functionally equivalent. HIF-1α is rapidly induced under acute hypoxia (0–24 h) and is widely expressed in HSCs, macrophages, and hepatocytes, where it drives the transcription of pro-fibrotic genes and glycolytic enzymes. In contrast, HIF-2α predominantly functions under chronic hypoxia (>24 h) and is preferentially upregulated in endothelial cells and HSCs, where it sustains HSC activation by regulating glutamine metabolism and the Yes-associated protein (YAP) signaling pathway [31]. This temporal and cell-type-specific divergence underlies their distinct roles in HSC activation and fibrotic progression, which are discussed in detail in Section 4.

## 4. HIF-Mediated HSC Activation: Molecular Mechanisms

HIFs drive HSC activation and liver fibrosis through five interconnected mechanisms: direct transcriptional regulation, synergy with TGF-β signaling, cross-talk with the NF-κB pathway, metabolic reprogramming, and paracrine networks. Before dissecting these individual mechanisms, it is important to address a fundamental controversy in the field: the distinct and, in some contexts, opposing roles of HIF-1α and HIF-2α in liver fibrosis. While HIF-α is widely recognized as a pre-fibrotic driver that promotes glycolysis, inflammation, and ECM deposition in HSCs, the function of HIF-2α is considerably more complex and context-dependent. On one hand, HIF-2α promotes liver fibrosis by sustaining HSC activation (e.g., through glutaminolysis and YAP signaling), inducing hepatocyte apoptosis and subsequent paracrine pro-fibrotic signals [24,32]. On the other hand, HIF-2α has also been reported to exert protective effects in certain contexts—particularly in macrophages, where it promotes an anti-inflammatory phenotype and its deficiency exacerbates NASH progression [33]. This functional dichotomy is further complicated by disease stage and model specificity: while hepatocyte-specific HIF-2α deletion attenuates fibrosis in dietary NASH models, it fails to confer protection in CCl_4_-induced toxic liver injury [24]. Rather than viewing HIF-2α as simply pro- or anti-fibrotic, it should be recognized as a context-dependent modulator whose net effect is determined by the specific cell type, disease stage, and microenvironmental milieu. This complexity has significant therapeutic implications, which will be revisited in Section 5 in the context of HIF-2α-selective targeting strategies.

### 4.1. Direct Effects on HSCs

HIF-1α is widely expressed in HSCs, macrophages, and hepatocytes. Under acute hypoxia (0–24 h), the stabilized HIF-1α subunit directly regulates the activation of HSCs. Mechanistically, HIF-1α depends on binding to the HRE in the promoter regions of target genes, thereby upregulating the transcription of pro-fibrotic genes such as *P4HA2* (a key enzyme for collagen synthesis) and chemokine receptors *CCR1* and *CCR5*, directly driving the activation, proliferation, and collagen synthesis of HSCs [26].

### 4.2. Interactions with TGF-β Signaling

The HIF-α pathway and the TGF-β/Smad (Sma- and Mad-related protein) signaling axis engage in extensive and intimate bidirectional crosstalk. HIF-1α serves both as an upstream transcriptional activator of TGF-β1 and as a partner in a potent synergistic regulatory loop with the TGF-β/Smad pathway. In turn, TGF-β1 activates hepatic stellate cells and upregulates alpha-smooth muscle actin (α-SMA) and collagen expression primarily through Smad2/3, and together these pathways drive liver fibrosis progression [34].

Under hypoxic conditions (1% O_2_), human dermal fibroblasts exhibit a marked upregulation of HIF-1α protein expression, accompanied by significant increases in the protein levels of TGF-β1, Smad2/3, phosphorylated Smad2/3 (p-Smad2/3), and Smad4. Immunofluorescence staining reveals that Smad4 is predominantly localized in the nucleus, suggesting its role as a transcription factor involved in downstream gene regulation. These findings indicate that hypoxia activates the TGF-β1/Smad signaling pathway via HIF-1α, thereby promoting collagen deposition [23,34].

In addition, HIF-1α not only directly activates target gene transcription but also enhances the DNA-binding capacity of Smad3. Phosphorylated Smad3 occupies the same gene loci together with HIF-1α, synergistically activating downstream genes, and amplifying hypoxia and TGF-β signals at the level of single gene sites. This synergistic mechanism leads to the dual activation of pro-fibrotic genes (such as *CTGF*, *COL1A1*) when hypoxia and TGF-β coexist, with expression levels far exceeding those of single stimulation, thereby accelerating liver fibrosis.

Moreover, *Smad4* forms a complex with *Smad2/3* and serves as a common mediator of TGF-β signaling. Knockdown of *Smad4* reduces both HIF-1α mRNA and protein levels, further suggesting reciprocal regulation between the two pathways. This points to the existence of a potential positive feedback loop in the hypoxia-HIF axis and TGF-β signaling, wherein HIF-1α upregulates Smad4 expression, which in turn further promotes HIF-1α expression, thereby amplifying pro-fibrotic signals under hypoxic conditions [35]. On the other hand, this reactive oxygen species (ROS)-mediated “oxidative hypoxia” establishes a self-amplifying loop involving NADPH oxidase (NOX), ROS, PHD2, and HIF-1α (i.e., a NOX-ROS-PHD2-HIF-1α loop) that persistently activates HSCs even under normoxia. [34].

### 4.3. Interaction with the NF-κB Inflammatory Pathway

Apart from interacting closely with the TGF-β/Smad pathway, HIF-1α also exerts pro-fibrotic effects through close association with NF-κB. These two molecules possess common oxygen-sensing elements and establish a positive feedback relationship. Such interaction jointly enhances metabolic adaptation and inflammatory reactions under hypoxia, which greatly facilitates liver fibrotic development.

IκB kinase (IKK) acts as a key kinase in NF-κB activation. Normally, IκBα binds NF-κB and retains it in the cytoplasm. Once phosphorylated by IKK, IκBα will be degraded, allowing NF-κB to become activated. PHD1 and PHD2 are critical upstream regulators controlling IKK function. Under normoxia, both proteins exert hydroxylase activity to hydroxylate IKKα and IKKβ, accelerating their proteasomal degradation and further suppressing NF-κB activity. Hypoxia suppresses the function of PHD1 and PHD2, which in turn causes intracellular accumulation of IKKα/β. Accumulated IKK proteins further induce IκBα degradation, promote NF-κB nuclear translocation and eventually trigger full NF-κB activation [36].

HIF-1α and NF-κB cross-regulate each other. Activated NF-κB not only transactivates inflammatory genes (including ACTA2 and COL1A1) and upregulates α-SMA and collagen protein levels, but also directly binds to the HIF1A promoter to drive HIF-1α transcription and activates anti-apoptotic genes (e.g., BCL2, BIRC3), thereby inhibiting HSC apoptosis [37,38]. Furthermore, HIF-1α induces pro-inflammatory cytokines such as IL-6 and TNF-α, which activate NF-κB, establishing a positive feedback loop between HIF-1α and NF-κB.

Beyond their mutual regulation, both HIF-1α and NF-κB are also modulated by the shared oxygen-sensing molecules PHD1 and PHD2, which thus serve as molecular hubs connecting hypoxia and inflammation in liver fibrosis [31,35]. Under normoxic conditions, TGF-β1 activates NOX to generate ROS, which oxidize the Fe^2+^ cofactor of PHD2, impairing its ability to hydroxylate HIF-1α and thereby causing aberrant stabilization of HIF-1α in a state termed “oxidative hypoxia” [34]. Simultaneously, hypoxia relieves the negative regulation of inhibitor of nuclear factor kappa-B kinase subunit beta (IKKβ) by PHDs, leading to IKKα/β phosphorylation, IκBα degradation, and enhanced DNA-binding activity of NF-κB [39]. The mitogen-activated protein kinase (MAPK) signaling pathway is activated under hypoxic conditions and further amplifies the transcriptional activities of HIF-1α and NF-κB through a phosphorylation cascade. Inactivation of PHD1/PHD2 results in HIF-1α stabilization, driving metabolic reprogramming and collagen synthesis; simultaneously, IKK escapes degradation, activating NF-κB [31,36]. Thus, through PHD1/PHD2, both the HIF-1α pathway (mediating metabolic adaptation) and the IKK/NF-κB pathway (mediating inflammation) are concurrently activated, leading to a synergistic amplification of these two responses.

The interactive feedback of HIF-1α and NF-κB aggravates pro-fibrotic activities. Hence, combined intervention against both pathways may be more effective than single pathway inhibition.

### 4.4. HIF-Driven Metabolic Reprogramming of Activated Hepatic Stellate Cells: Glycolysis and Glutamine Metabolism

Apart from regulating gene transcription to activate HSCs, HIF-α also induces adaptive metabolic reprogramming such as glycolysis and glutamine metabolism. These metabolic changes meet the elevated energy and biosynthesis needs when quiescent HSCs transform into activated myofibroblasts [18,40]. HIF-1α and HIF-2α regulate different yet mutually supportive metabolic pathways in the hypoxic microenvironment [40].

In acute hypoxia, HIF-1α primarily mediates the classical Warburg effect. It upregulates the expression of key glycolytic genes including *GLUT1* and *PFKFB3*, which in turn enhance glucose uptake and overall glycolytic rate. Meanwhile, HIF-1α increases pyruvate kinase M2 (PKM2) and lactate dehydrogenase A (LDHA), and upregulates pyruvate dehydrogenase kinase 1 (PDK1) to inhibit pyruvate from entering the tricarboxylic acid (TCA) cycle. This process enables cells to develop aerobic glycolysis features even under normoxic conditions. Enhanced glycolysis accelerates HSC activation and provides sufficient energy as well as synthetic raw materials for ECM deposition. Additionally, lactate buildup in the local microenvironment further facilitates *HIF1A* transcription, ultimately forming a positive feedback loop.

Recent studies point to other regulatory layers. Under normoxia, Wnt/β-catenin signaling sends LDHA into the nucleus, where it teams up with HIF-1α to form a transcriptional complex. By covering the oxygen-dependent degradation domain of HIF-1α, LDHA blocks PHD2-mediated hydroxylation. This prevents proteasomal degradation of HIF-1α and makes it more stable. The Gly28 residue of LDHA is key to forming the LDHA/HIF-1α complex, which then turns on glycolytic genes like hexokinase 2 (*HK2*) and phosphofructokinase-1 (*PFK1*), sustaining the aerobic glycolysis phenotype [18]. Also, in human liver fibrosis samples and mouse models induced by carbon tetrachloride (CCl_4_) or bile duct ligation (BDL), HIF-1α accumulation drives the transcription of glycolytic genes including *SLC2A1*, *HK2*, and *SLC16A3*, thereby promoting aerobic glycolysis in activated HSCs [31].

Unlike HIF-1α, which acts as the “first responder” during hypoxia, HIF-2α functions predominantly under chronic hypoxia (>24 h) and serves as a sustainer of HSC activation and metabolic reprogramming. HIF-2α is predominantly upregulated in endothelial cells and HSCs under prolonged hypoxia, with greater protein stability [31]. The transcription factor GATA binding protein 4 (GATA4) directly represses *EPAS1* (endothelial PAS domain protein 1) in quiescent HSCs; upon GATA4 downregulation, HIF-2α accumulates, explaining the delayed activation of HIF-2α relative to HIF-1α [41].

Under chronic hypoxia, HIF-2α selectively activates HSCs by promoting YAP nuclear translocation through the inhibition of YAP phosphorylation at Ser127. This leads to upregulation of glutaminase 1 (GLS1), enhancing glutamine metabolism and providing additional energy support for persistently activated HSCs [32,33,42]. This glutamine-dependent metabolic shift works alongside HIF-1α-driven glycolysis; together, they meet the high energy demands of activated HSCs. Moreover, activated HSCs release exosomes loaded with glycolytic proteins such as GLUT1 and PKM2. Once taken up by quiescent HSCs, Kupffer cells (KCs), and LSECs, these exosomes transfer a glycolytic (Warburg) phenotype to the recipient cells, creating a form of metabolic synergy that accelerates liver fibrosis. Disrupting HIF-1α expression blocks this exosome-mediated glycolytic transfer [43].

### 4.5. Hepatocyte HIFs and Paracrine HSC Activation

Paracrine signals originating from hepatocytes and macrophages under hypoxia also play a non-negligible role in promoting liver fibrosis. Under hypoxia, hepatocytes activate HIF-1α and HIF-2α, secreting pro-fibrotic factors, including TGF-β1 and VEGF. These factors act in a paracrine manner on HSCs, stimulating their activation and EMT. For instance, LTBP2 is a paracrine factor derived from hepatocytes. Its transcription is directly upregulated by hepatocyte HIF-1α. This factor then activates the extracellular signal-regulated kinase (ERK) signaling pathway in HSCs. As a result, it promotes HSC activation, proliferation, migration, and EMT [44]. HSC-specific knockdown of *LTBP2* significantly eases CCl_4_-induced liver fibrosis in mice, confirming LTBP2 as a key paracrine effector downstream of hepatocyte HIF-1α.

Using the CCl_4_-induced mouse model of liver fibrosis, researchers find that HIF-2α is predominantly expressed in hepatocytes. Hepatocyte-specific *Hif-2α*-knockout reduces apoptosis and consequent pro-fibrotic paracrine signals, leading to decreased HSC activation and fibrosis without affecting inflammation. Additionally, HIF-2α deficiency upregulates hepatocyte-specific transcription factors (e.g., *HNF4α*) and lipid metabolism genes (e.g., *PPARα*, *CPT1α*), preserving hepatocyte function and indirectly limiting fibrogenic paracrine crosstalk. These findings suggest that paracrine signals from hepatocytes, orchestrated by HIF-1α and HIF-2α, are critical drivers of HSC activation and liver fibrosis [24].

Hypoxic hepatocytes also release small extracellular vesicles (sEVs), enriched with mannose-binding lectin-associated serine protease 1 (MASP1) from hepatocytes, by regulating the autophagy-lysosome/multivesicular body (MVB) pathway and Rab27A. Once secreted, these MASP1-carrying sEVs activate HSCs via p38 MAPK/ATF2 signaling, which in turn speeds up liver fibrosis [45].

**Figure 2:**
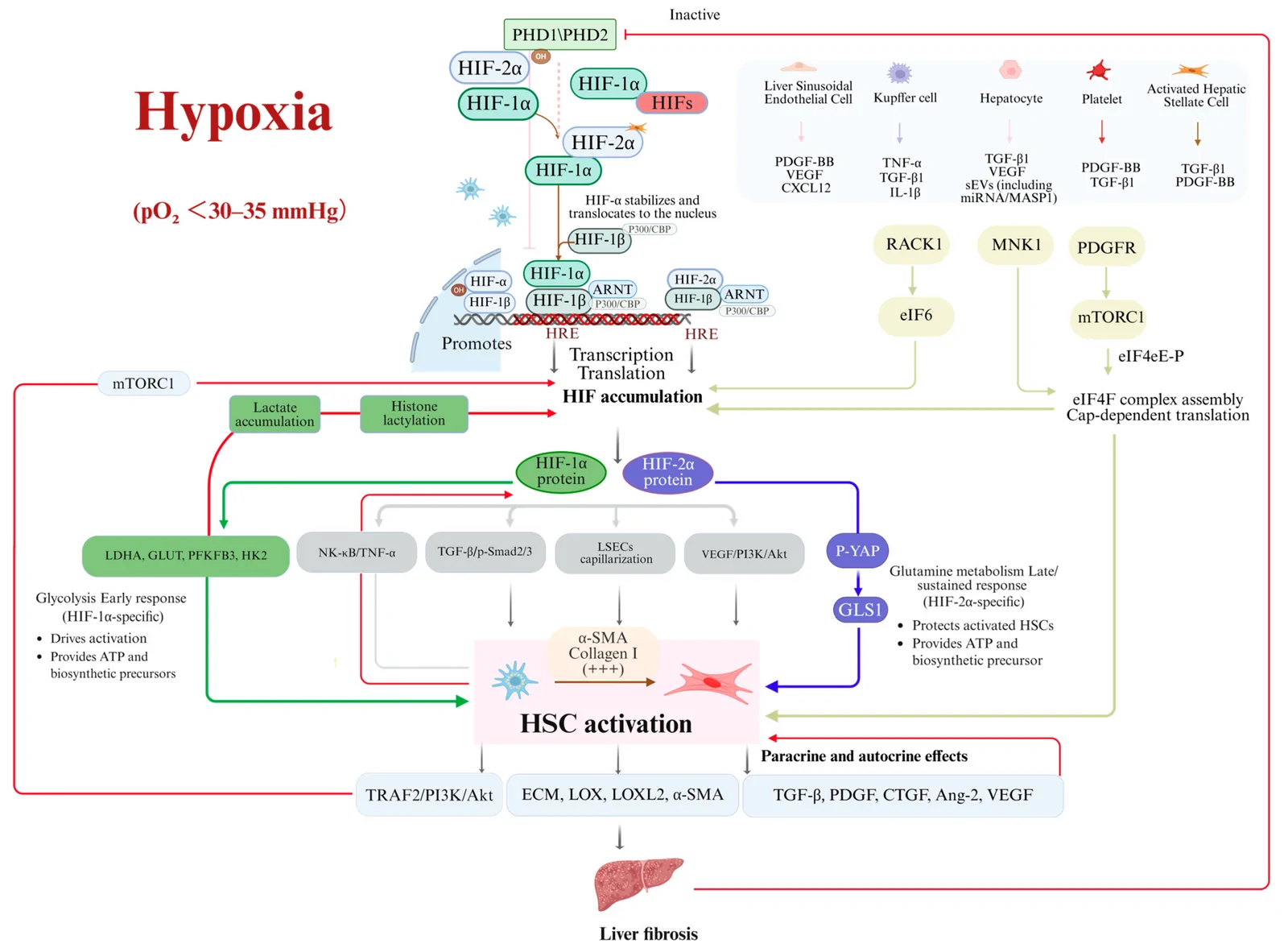
Hypoxia-driven hepatic stellate cell activation and signaling networks underlying liver fibrosis. Under physiological conditions, hepatic oxygen homeostasis is maintained by sinusoidal blood flow, intact LSECs, and quiescent HSCs. In chronic liver injury, reduced oxygen tension stabilizes hypoxia-inducible factors (HIF-1α and HIF-2α) by inhibiting PHD1/PHD2, thereby promoting HIF nuclear translocation and transcriptional activation. Hypoxia-induced signaling is further amplified by inflammatory mediators released from hepatocytes, Kupffer cells, platelets, and LSECs, including TNF-α, TGF-β, PDGF, VEGF, IL-1β, IL-6, and CXCL12. These factors, together with hypoxia-associated metabolic reprogramming, lactate accumulation, histone lactylation, mTORC1 activation, VEGF/PI3K/Akt signaling, and P-YAP/GLS1-mediated glutaminolysis, converge to promote HSC transdifferentiation into proliferative, contractile, myofibroblast-like cells characterized by α-SMA expression and excessive extracellular matrix production, including collagen type I, LOX/LOXL2, and other ECM components. In parallel, hypoxia induces LSEC capillarization, fenestrae loss, and basement membrane deposition, further exacerbating sinusoidal dysfunction and microcirculatory impairment. Sustained HSC activation establishes autocrine and paracrine profibrogenic loops, driving progressive hepatic fibrosis and ultimately cirrhosis. In the fibrotic liver, the resulting hypoxic microenvironment stabilizes HIF-1α and HIF-2α via inactivation of PHDs, which normally target HIF-α for VHL-mediated proteasomal degradation. Stabilized HIF-α translocates to the nucleus, dimerizes with HIF-β, and transactivates a broad repertoire of target genes. In parallel, HIF-α signaling converges on the translational machinery in HSCs through multiple nodes: mTORC1-dependent phosphorylation of 4E-BP1 relieves its inhibitory binding to eIF4E. This event collectively promotes eIF4F complex assembly and cap-dependent translation of pro-fibrotic mRNAs (e.g., *COL1A1*, *Snail*, and *cyclin E1*). Additionally, eIF6, which is upregulated by RACK1 in fibrotic livers, facilitates 60S ribosomal subunit biogenesis and selectively drives C/EBPβ-LIP translation, sustaining the metabolic reprogramming required for HSC proliferation and ECM production. The integrated HIF-translational cascade ultimately activates HSCs, conferring a myofibroblastic phenotype characterized by enhanced proliferation, migration, contractility, survival, and ECM deposition, thereby propelling the progression from fibrosis to cirrhosis and HCC. Moreover, these mechanisms constitute a self-sustaining cycle of hypoxia, HSC activation, and ECM deposition, driving the progression of liver fibrosis. Solid arrows indicate direct activation or conversion; dashed arrows represent reaction pathways that occur under normal conditions; and T-shaped bars indicate inhibition. Abbreviations are defined in the main text and the abbreviation list. Created in BioRender. LI, W. (2026). (For computer program and AI disclosure, see the statement preceding the figures.)

Kupffer cells are the resident liver macrophages. Under hypoxia, Kupffer cells adopt a pro-fibrotic phenotype and activate HSCs via a TGF-β-dependent mechanism. HIF-2α in hepatic macrophages exerts pro-inflammatory effects by promoting KC death and recruiting pro-inflammatory macrophages, thereby driving NASH progression. Interestingly, HIF-1α and HIF-2α have opposite roles in macrophages: HIF-1α promotes pro-inflammatory M1 polarization, whereas HIF-2α is usually considered an anti-inflammatory M2 polarization promoter. However, under NASH conditions, HIF-2α exhibits pro-inflammatory properties instead, suggesting disease-specific regulation. The comprehensive hypoxia-initiated signaling network governing HSC activation during liver fibrogenesis described above is schematically illustrated in Figure 2.

## 5. Therapeutic Targeting of Hypoxic Pathways in Liver Fibrosis

Given the central role of the HIF axis in liver fibrosis, targeting this axis has emerged as a promising therapeutic strategy. Current research mainly focuses on small-molecule inhibitors of HIFs, and agents targeting its upstream regulators or downstream key effector molecules.

### 5.1. Small-Molecule HIF Inhibitor Subsection

Several small-molecule inhibitors of HIF-α 1 have been identified in multiple studies, which can be classified into three categories based on their targets.

#### 5.1.1. HIF-1α Inhibitor

Small-molecule HIF-1α inhibitors alleviate liver fibrosis by blocking HIF-1α transcriptional activity or promoting its degradation. Digoxin and its analogs have been widely used as cardiac drugs in clinical practice. They have recently been found to possess potent HIF-1α inhibitory activity, offering a potential drug-repurposing opportunity for liver fibrosis treatment [46]. Digoxin prevents PKM2 from nuclear translocation and interaction with histone H3, thereby inhibiting PKM2-mediated HIF-1α transcriptional activation. Digoxin (1 mg/kg, intraperitoneal injection twice weekly) significantly downregulated *Vegf*, *Glut1*, and *Il1b*, and reduced hepatic steatosis, inflammatory cell infiltration, and hepatocyte injury in a NASH model. Beyond that, these protective effects were abolished in HIF-1α conditional knockout mice, suggesting that HIF-1α is a key target mediating digoxin’s hepatoprotective effect [46].

Unlike digoxin, there is currently no direct evidence that echinocandins exert anti-liver-fibrotic effects through HIF-1α inhibition. Existing studies mainly focus on the involvement of HIF-1α in the hepatotoxic mechanisms of these drugs rather than their therapeutic applications. Caspofungin, an antifungal drug, has been reported to modulate HIF-1α signaling in the context of drug-induced liver injury, but its direct anti-fibrotic potential remains speculative and requires further investigation [47].

One of the most advanced anti-fibrotic candidates for liver fibrosis is AKF-PD (fluorofenidone), which entered a Phase II clinical trial (approval number: 2016L09979) as a Class 1.1 new drug in China in 2016. AKF-PD is a novel low-molecular-weight pyridine-based anti-fibrotic compound developed independently in China. Recent mechanistic studies have shown that AKF-PD induces ferroptosis in HSCs by specifically inhibiting the HIF-1α/solute carrier family 7 member 11 (SLC7A11, also known as xCT) pathway, thereby exerting anti-fibrotic effects [48]. In a CCl_4_-induced rat model of liver fibrosis, AKF-PD (120–240 mg/kg) dose-dependently reduced extracellular matrix deposition by suppressing HIF-1α protein expression, which in turn downregulated SLC7A11 and glutathione peroxidase 4 (GPX4). This led to glutathione depletion, lipid reactive oxygen species (ROS) accumulation, and iron-dependent cell death in HSCs. Notably, HIF-1α overexpression partially reversed the pro-ferroptotic effect of AKF-PD, whereas HIF-1α knockdown synergized with AKF-PD to amplify its anti-fibrotic action, further confirming that HIF-1α is a key target of this compound [48].

YC-1 (lificiguat) is one of the most extensively studied small-molecule inhibitors of HIF-1α. Research has demonstrated that this compound suppresses HIF-1α activity under hypoxic conditions in five types of tumor cells, including Hep3B hepatocellular carcinoma, and affects both the protein itself and its downstream genes (*VEGF*, aldolase, enolase) [49]. In the related research on the tension of isolated mouse aortic rings, it was confirmed that YC-1 has a direct activating effect on sGC [50]. This indicates that YC-1 may exert anti-fibrotic effects through the dual mechanisms of inhibiting the expression of HIF-1α protein to block the generation and angiogenesis of VEGF induced by hypoxia and activating soluble guanylate cyclase (sGC), thereby regulating vascular tension. A 2023 study further revealed that YC-1 inactivates the NF-κB/HIF-1α pathway in liver tissue of CCl_4_-induced fibrotic rats and in CoCl_2_-induced hypoxic RAW264.7 cells, thereby inhibiting M1 macrophage polarization and alleviating hepatic inflammation and fibrosis, positioning YC-1 as a potential drug for liver fibrosis [37]. Paeoniflorin, acting analogously to YC-1, reduces p65 and IκBα phosphorylation and HIF-1α expression, decreases inflammatory cell infiltration and collagen deposition in liver tissue, suppresses HSC activation (as evidenced by reduced α-SMA), and attenuates extracellular matrix deposition, thereby mitigating liver inflammation and fibrosis and offering a potential therapeutic target for liver fibrosis [37]. Similarly, the novel HIF-1α inhibitor AMSP-30m has been shown to attenuate CCl_4_-induced liver fibrosis in mice by suppressing the Sonic Hedgehog (Shh) pathway [51].

These findings suggest that direct pharmacological inhibition of HIF-1α may represent a feasible anti-fibrotic strategy. Nevertheless, such a strategy warrants caution: HIF-1 serves as a central orchestrator of whole-body oxygen sensing and homeostasis. Moreover, HIF-1α and HIF-2α exhibit functionally opposing roles—particularly under conditions of chronic activation—where sustained HIF-1α signaling exacerbates oxidative damage, inflammatory responses, and extracellular matrix deposition, whereas HIF-2α often acts to dampen these pathological processes. In models of chronic cardiovascular, renal, and hepatic disorders, a pathogenic shift—characterized by hyperactive HIF-1α coupled with suppressed HIF-2α activity—has been consistently linked to disease worsening [52]. This functional dichotomy may stem from cell-context-dependent actions of HIF-1α; while it drives fibrogenesis in activated hepatic stellate cells, it may concurrently mediate cytoprotective or anti-inflammatory functions in hepatocytes or specific immune subsets. Therefore, systemic or non-cell-type-specific inhibition of HIF-1α could lead to adverse consequences. Targeting HIF-2α or other pathways may offer a more favorable therapeutic strategy. The small-molecule HIF inhibitors discussed in this review are summarized in Table 1.

#### 5.1.2. HIF-2α Selective Antagonist

The relative contributions of HIF-1α and HIF-2α to liver fibrosis vary with disease stage. HIF-1α responds to acute cellular hypoxia, whereas HIF-2α mainly sustains HSC activation and drives disease progression under chronic hypoxia. Therefore, selective targeting of different HIF isoforms may be required at different fibrotic stages, with HIF-2α selective antagonists offering a greater advantage in mid-to-late stage liver fibrosis. Several HIF-2α selective antagonists (e.g., PT2385, belzutifan) have been approved for specific cancers. Based on evidence that HIF-2α drives fibrosis in HSCs in chronic metabolic injury models, these agents also show significant therapeutic promise for NAFLD/NASH [24].

Belzutifan is the first FDA-approved HIF-2α-selective inhibitor for the treatment of VHL disease-associated renal cell carcinoma [53]. It specifically binds to the PAS-B domain of HIF-2α, blocking its heterodimerization with ARNT and thereby inhibiting HIF-2α target gene transcription [54]. Based on the critical role of HIF-2α in NASH fibrosis, belzutifan has a clear theoretical rationale for application in liver fibrosis. Studies have shown that HIF-2α inhibits YAP phosphorylation and promotes its nuclear translocation, thereby enhancing glutamine catabolism in HSCs and providing energy for myofibroblast activation [32,55,56]. HIF-2α also promotes collagen deposition by increasing the expression of fibrotic markers such as α-SMA and Col1A [24,57]. Theoretically, by suppressing YAP-mediated glutamine metabolism and collagen synthesis, HIF-2α inhibition could block persistent HSC activation.

Although there is theoretical potential for alleviating liver fibrosis, studies on the application of HIF-2α-selective inhibitors in liver fibrosis remain limited. In addition, given that HIF-2α may act as an antagonist to HIF-1α in certain models, selective inhibition of HIF-2α could theoretically lead to compensatory activation of the HIF-1α pathway, potentially exacerbating fibrosis. Therefore, targeting HIF-2α alone as a therapeutic strategy for liver fibrosis also faces considerable constraints.

#### 5.1.3. Dual-Target HIF Inhibitor Subsubsection

HIF-1α and HIF-2α have distinct functions in liver fibrogenesis and can compensate for each other. As a result, direct inhibition of a single isoform often yields limited efficacy [58]. Dual-target inhibitors that simultaneously target both HIF-1α and HIF-2α may achieve better therapeutic outcomes. A 2024 study identified that an HIF microcycle binds to the PAS-B domain of HIF-α, overlapping with the key binding loop of HIF-1β. As a result, the protein–protein interaction between the α and β subunits was disrupted, and the function of both HIF-1 and HIF-2 transcription factors was impaired [59].

### 5.2. Targeting HIF Upstream Regulators

Therapeutic approaches that target the upstream regulatory mechanisms of HIF-α may offer unique advantages compared to selective HIF-1α or HIF-2α inhibitors or dual-targeting strategies, although careful evaluation is still needed. Targeting upstream of HIF-α not only eliminates the stability of both HIF-1α and HIF-2α proteins, thereby preventing isoform-compensatory activation, but also acts on upstream modification nodes involved in protein stabilization, potentially avoiding adaptive mutations and off-target toxicity at downstream functional sites. Evidence from animal models suggests that *VHL* overexpression exhibits superior anti-fibrotic effects compared to combined gene silencing, supporting the translational potential of upstream-targeting.

However, the cell-type-dependent functions of HIF-α must be considered. While HIF-1α promotes fibrosis in HSCs, it may exert adaptive protective effects in hepatocytes (e.g., metabolic reprogramming, anti-oxidative stress) and modulate inflammatory responses in Kupffer cells. Thus, under certain circumstances, upstream inhibition could lead to excessive inflammation, hepatocyte injury, or immune dysregulation. Furthermore, the treatment of liver fibrosis may require a staged approach considering the functional divergence between HIF-1α and HIF-2α. Upstream regulation seems to be more effective in acute injury, because hypoxia and HIF-α activation are early events. However, in established fibrosis, downstream signals (e.g., TGF-β1, CTGF) are already activated, which may sustain themselves through autocrine/paracrine loops, instead of being fully dependent on HIF-α. In this scenario, targeting direct effectors downstream of HIF-α, such as VEGF or LOX, might provide more targeted intervention. Therefore, upstream regulation should be considered as part of a stratified strategy rather than absolute superiority.

#### 5.2.1. Bidirectional Regulation of PHD Activity

The degradation rate of HIF-α under normoxic conditions is directly determined by the activity of PHD enzymes. However, as oxygen-sensitive molecules, PHD activity is not only influenced by oxygen concentration but also finely regulated by the ROS microenvironment. In the fibrotic microenvironment, reduced oxygen levels and excessive ROS production lead to iron ion oxidation in the catalytic center of PHD2, causing its inactivation. This allows HIF-1α to stabilize and translocate to the nucleus, driving pathological effects [34]. In a bleomycin-induced pulmonary fibrosis model, ACF-2 specifically binds to PHD2 and scavenges ROS in its microenvironment, thereby restoring the hydroxylase activity of PHD2 and effectively blocking the aforementioned positive feedback loop. This compound exhibited superior anti-fibrotic effects compared to nintedanib [34]. These findings provide a new strategy for treating liver fibrosis by restoring PHD function.

#### 5.2.2. Inhibition of HIF-α Transcription and Translation: mTOR Pathway Blockade

In the chronic liver disease microenvironment, the mTOR signaling pathway, as the core hub of cell metabolism and growth, plays a crucial role in the occurrence and development of liver diseases. The abnormal activation of mTOR signaling has a significant impact on liver diseases. mTOR forms two functionally distinct complexes, mTORC1 and mTORC2, of which mTORC1 is sensitive to rapamycin [60].

mTORC1 is a key regulatory hub for the translation and synthesis of HIF-1α protein. On one hand, under hypoxic conditions, the stable accumulation of HIF-1α mainly depends on the inactivation of oxygen-dependent PHD, but the final protein level of HIF-1α is also significantly influenced by translation efficiency. mTORC1 enhances ribosome biogenesis and 5′ cap-dependent translation initiation by phosphorylating S6K and 4E-BP1, thereby directly increasing the protein synthesis efficiency of HIF-1α mRNA and promoting lipid production, which accelerates the progression of non-alcoholic fatty liver disease. In the context of liver fibrosis, the continuous activation of the mTORC1 signaling pathway provides necessary anabolic support for the activation and proliferation of HSCs [61]. In research on the role of the LKB1-AMPK pathway in various tumors, it was found that when cells are under energy stress, AMPK, as an energy sensor, is activated. It directly inhibits the activity of mTORC1 by phosphorylating TSC2 and raptor, thereby reducing the translation synthesis of HIF-1α [62]. Therefore, mTORC1 integrates multiple signals from growth factors, nutrients, oxygen supply, and energy status, becoming the core regulatory node for the protein level of HIF-1α. Its activity changes directly affect the translation output of HIF-1α [61].

Prior studies have established tumor necrosis factor receptor-associated factor2 (TRAF2) as a critical upstream adaptor in TNF-α-induced NF-κB signaling during inflammatory responses. In the context of hypoxia, activation of the mTORC1 pathway has been shown to promote HIF-1α translation in HSCs, thereby enhancing glycolytic metabolism and sustaining HSC activation [63,64].

After HIF-1α stabilizes and translocates to the nucleus, its subsequent transcription and translation are highly dependent on the upstream PI3K/AKT/mTOR signaling pathway. In early hypoxia, upstream signals of HSC activation such as PDGF-BB, VEGF, and TRAF2 activate the PI3K/AKT/mTORC1 axis, promoting HIF-1α mRNA translation. Rapamycin, as a non-covalent inhibitor of mTORC1, can block the phosphorylation of S6K and 4E-BP1 induced by overexpression of TRAF2, thereby effectively inhibiting the increase in HIF-1α translation dependent on mTORC1, reducing the protein level of HIF-1α and its downstream GLUT1-mediated glycolytic activity, and ultimately inhibiting the activation of HSCs and alleviating liver fibrosis [65]. Moreover, this drug repurposing strategy offers a relatively rapid path toward clinical translation. Given that rapamycin and its derivatives (everolimus, sirolimus, etc.) have been widely used as immunosuppressants in clinical practice, using them as anti-fibrotic drugs may have a faster clinical translation path compared to the development of completely new drugs. However, it should be noted that the inhibition of mTORC1 by rapamycin is incomplete—4E-BP1 phosphorylation is relatively insensitive to rapamycin, and long-term systemic mTOR inhibition may cause metabolic disorders, immune suppression and other adverse reactions. Therefore, developing more HSC-targeted mTORC1 intervention strategies or combining with other anti-fibrotic targets may be a future research direction.

#### 5.2.3. Blocking Upstream Inflammatory Signals: Targeting the TRAF2/NF-κB Axis

In the progression of liver fibrosis, TRAF2 acts as an upstream driver of the HIF-1α/glycolysis axis. TRAF2 represents a central hub that integrates inflammatory signaling, metabolic reprogramming, and fibrotic cascades. TRAF2 not only inhibits pVHL-mediated ubiquitin-proteasome degradation of HIF-1α and promotes HIF-1α translation via the PI3K/AKT/mTORC1 signaling pathway, but it also enhances glycolysis by activating PFKFB3. Moreover, TRAF2 upregulation phosphorylates IKK, leading to the degradation of IκBα that sequesters NF-κB in the nucleus, thereby promoting NF-κB release. Through these multifaceted actions, TRAF2 influences HIF-1α stabilization/nuclear translocation, HIF-1α translation, glycolysis, and the NF-κB signaling pathway simultaneously. Therefore, directly targeting TRAF2 or its downstream effectors suppresses HIF-1α expression and glycolysis, as well as blocking NF-κB-mediated release of inflammatory cytokines, representing an effective strategy to interrupt the vicious cycle linking inflammation, metabolic reprogramming, and fibrosis [61]. Just as we had suspected, experiments have already proven that specific knockout of TRAF2 in HSCs significantly alleviates liver injury and fibrosis in mice, further confirming the central role of the TRAF2/HIF-α axis in the activation of HSCs [26]. Thus, a single agent that specifically inhibits TRAF2 could achieve multi-target intervention.

### 5.3. Targeting HIF Transcriptional Targets

#### 5.3.1. Vascular Endothelial Growth Factor Receptor (VEGFR) Antagonist

Through HIF-1α, the hypoxic microenvironment strongly upregulates VEGF expression, promoting pathological angiogenesis. VEGF also exerts a paracrine action on HSCs by activating mTOR to enhance HIF-1α translation and HIF-2α transcription [66,67,68]. Studies have shown that HSCs express VEGFR-2, and VEGF binding to this receptor activates the phospholipase C-gamma (PLC-γ)/ERK signaling pathway, directly promoting HSC proliferation and migration [69,70]. PTK/ZK, an oral, potent angiogenesis inhibitor, is characterized by its ability to simultaneously inhibit all known VEGF receptor tyrosine kinases, including VEGFR-1 (*Flt-1*), VEGFR-2 (*KDR/Flk-1*), and VEGFR-3 (*Flt-4*), with the strongest inhibitory activity against VEGFR-2. Therefore, the use of tyrosine kinase inhibitors (e.g., sorafenib) or specific monoclonal antibodies targeting this receptor axis may suppress hypoxia-induced HSC activation by blocking the PLC-γ/ERK paracrine loop. As a result, hepatic microvascular density would be reduced, and fibrotic progression would be retarded [71]. This suggests that targeting the VEGF/VEGFR axis directly curbs stromal cell activation on top of inhibiting angiogenesis.

Furthermore, PTK/ZK also exhibits some inhibitory activity against other tyrosine kinases that belong to the same family as VEGFR, including PDGFR-β, c-kit, and c-fms [72]. Simultaneously, PTK/ZK reduces TGF-β receptor (TGFβRI/II) expression, suppresses TGF-β1-induced expression of VEGF and VEGFR1, and blocks the phosphorylation of Akt, ERK, and p38 MAPK [72]. Thus, by concurrently targeting the PDGF and TGF-β signaling pathways, PTK/ZK achieves dual inhibition of HSC activation.

#### 5.3.2. LOX Inhibitor

In addition to VEGF, LOX expression is also significantly upregulated in HSCs under hypoxic conditions. LOX and its isoforms (LOXL1–LOXL4), as direct target genes of HIF-1α, catalyze the oxidative deamination that forms covalent crosslinks between collagen and elastin within ECM, rendering deposited collagen fibers dense and resistant to degradation [73,74,75]. This “matrix stiffening” not only maintains the structural stability of the fibrotic scaffold but also feeds back through integrin signaling to enhance HSC contractility and sustained activation [74,76]. Therefore, inhibiting LOX activity may soften the fibrotic matrix and increase ECM degradability.

Given the central role of LOX in stabilizing deposited ECM, LOX inhibitors may have synergistic effects when combined with strategies that promote ECM degradation (e.g., matrix metalloproteinase activators or anti-inflammatory therapies) or with specific antibodies. For example, β-aminopropionitrile (BAPN, a LOX activity inhibitor) combined in treatment with resveratrol (RES) reduces cross-linking and attenuates fibrotic stiffness in CCl_4_-treated rats [77]. BAPN also inhibited the accumulation of crosslinked collagen in a BDL-induced liver fibrosis model [75]. Furthermore, a LOXL2-neutralizing antibody was shown to target extracellular LOXL2, promoting the recruitment of restorative monocyte-derived macrophages (MoMFs) to fibrotic areas and inducing secretion of matrix metalloproteinases (especially MMP-14). This altered collagen structure and accelerated collagen degradation, thereby improving liver fibrosis regression [78].

#### 5.3.3. Targeting Metabolic Reprogramming

As detailed in Section 4.4, HIF-1α drives aerobic glycolysis (via GLUT1, PFKFB3, LDHA, HK2) and HIF-2α drives glutaminolysis (via GLS1) to support HSC activation and ECM deposition. These metabolic dependencies offer druggable nodes for anti-fibrotic intervention.

The small-molecule PFKFB3-antagonist 3PO reduces HSC activation and proliferation. Knockdown of *PFKFB3* in HSCs alleviates the severity of liver fibrosis in mice. Mechanistically, *PFKFB3* upregulation in activated HSCs is mediated by cytoplasmic polyadenylation element binding protein 4 (CPEB4) binding to the 3′-untranslated region (3′-UTR) of its mRNA. Targeting the CPEB4-PFKFB3 axis may represent a novel therapeutic strategy [79]. HSC-specific knockdown of *LDHA* attenuates HSC activation and liver fibrosis in mice [18]. Canonical Wnt signaling activates *LDHA* transcription via β-catenin/TCF4, promoting its nuclear translocation and complex formation with HIF-1α. This complex stabilizes HIF-1α, enhances glycolysis, and ultimately drives HSC activation and liver fibrosis [18]. HK2, a rate-limiting enzyme of glycolysis, also plays a key role in aerobic glycolysis. A 2025 study by Han et al. using multi-omics integrative analysis revealed that HK2 expression is significantly upregulated in HSCs, leading to lactate accumulation, which alters histone pH and promotes HIF-1α transcription, and is closely associated with the progression of liver fibrosis [80]. Dichloroacetate (DCA) reverses the Warburg effect by inhibiting PDK, relieving phosphorylation-dependent inhibition of the pyruvate dehydrogenase complex (PDC). This shifts metabolism from glycolysis to glucose oxidation by promoting pyruvate entry into the TCA cycle, reducing lactate production, and improving tissue oxygenation—indirectly affecting the HIF-1α pathway [81,82]. In a mouse model of alcohol-associated liver disease (ALD), DCA also exhibited anti-inflammatory effects and ameliorated ethanol-induced hepatic metabolic dysregulation [83].

*GLS1* is a direct target of HIF-2α. Inhibition of HIF-2α or its downstream effector *YAP* reduces HSC activation and myofibroblast levels [84]. Urolithin A (UroA), a gut microbial metabolite and GLS1 inhibitor, directly binds to GLS1 at residues D327 and Y394, inhibiting its activity. This suppresses HSC activation, proliferation, and collagen synthesis, and significantly reduces portal pressure, liver fibrosis, ascites, and splenomegaly in a mouse model of cirrhotic portal hypertension (CPH) [19]. By targeting GLS1, UroA inhibits HSC glutaminolysis and alleviates intrahepatic vascular resistance, providing a new metabolic target and candidate drug for CPH.

In summary, targeting key nodes of glycolysis (PFKFB3, LDHA, DCA) and glutaminolysis (GLS1) effectively suppresses HSC activation and alleviates liver fibrosis, validating the anti-fibrotic potential of a “metabolic deprivation” strategy.

### 5.4. Nucleic Acid-Based Strategies

Although small-molecule inhibitors have shown promise in anti-fibrotic therapy, their off-target effects and systemic toxicity remain major challenges. In recent years, nucleic acid-based gene silencing strategies have provided new avenues for precisely targeting the HIF pathway.

RNA interference technology, particularly small interfering RNA (siRNA), offers a precise strategy for specifically silencing HIF-1α expression. By directly degrading HIF-1α mRNA, siRNA blocks hypoxic signal transduction at its source without affecting the function of other isoforms such as HIF-2α. siRNA targeting HIF-1α has been evaluated in preclinical fibrosis models. Studies have shown that liposome- or nanocarrier-mediated delivery of HIF-1α-specific siRNA significantly reduces HIF-1α protein levels in liver tissue. The anti-fibrotic mechanism primarily involves direct regulation of HSCs. Silencing HIF-1α blocks the expression of downstream glycolytic enzymes (e.g., PDK1) and pro-fibrotic factors (e.g., VEGF, TGF-β1). This not only inhibits glycolytic metabolic reprogramming in HSCs but also reduces ECM deposition, thereby significantly improving liver histopathological scores and fibrotic area.

MicroRNAs (miRNAs) are endogenous non-coding RNAs that regulate gene expression. Several miRNAs have been shown to target HIF-1α or its related pathways, thereby inhibiting HSC activation [85]. For instance, *miR-125a-5p* has been proven to suppress HSC activation and liver fibrosis via the TGF-β/Smad2/3 signaling pathway. The expression of this miRNA is significantly downregulated in activated HSCs and fibrotic liver tissues. Mechanistically, miR-125a-5p directly inhibits TGFβR1 and p-Smad2/3 by targeting TGFβR1, blocking the signal transduction of HSC activation. Also, miR-125a-5p significantly reduces the levels of α-SMA, Collagen I, and the proteins in the TGF-β/Smad2/3 pathway [86]. This in turn modulates the downstream TGF-β/Smad2/3 signaling pathway, resulting in inhibition of HSC activation and attenuation of liver fibrosis both in vitro and in vivo [86].

### 5.5. Translational Control in HSC Activation: Selective Synthesis of Pro-Fibrotic Proteins Under Hypoxia

HSC activation is the core driver of liver fibrogenesis, and this process is highly dependent on translational reprogramming. In the fibrotic liver, the hypoxic microenvironment stabilizes HIF-1α; however, the pro-fibrotic effects of HIF-1α require the coordination of the translational machinery. Studies have shown that eukaryotic translation initiation factor 4E-binding protein 1 (4EBP1) and eukaryotic initiation factor 6 (eIF6) play regulatory roles in the function of activated HSCs. In experiments using macrophage-conditioned medium (MCM) to stimulate HSC activation, receptor for activated C kinase 1 (RACK1) was found to simultaneously drive the mTORC1 and MNK1–eIF4E axes (directly phosphorylating eIF4E to enhance its cap-binding ability) [87]. These two pathways converge to promote eIF4E phosphorylation and eIF4F complex assembly, thereby selectively upregulating the translation of pro-fibrotic factor mRNAs that possess G+C-rich 5′-UTRs—including *COL1A1*, *Snail*, and *cyclin E1*—ultimately leading to HSC activation and liver fibrosis [87]. In addition, TGF-β can phosphorylate 4EBP1 via mTORC1, relieving its inhibitory effect on the translation initiation function of eukaryotic translation initiation factor (4EeIF4E) [88]. Notably, 4EBP1 acts as a translational brake, and its phosphorylation-mediated inactivation serves as a critical switch for HSC activation: the non-phosphorylatable 4EBP1 mutant (4EBP1-4A) not only downregulates HIF-1α protein levels but also directly suppresses HSC proliferation, migration, and collagen synthesis [88]. Regarding eIF6, a study investigating its role in driving NAFLD progression to HCC through translational control revealed that eIF6 selectively drives the translation of the C/EBPβ-LIP isoform by regulating 60S ribosomal subunit assembly, thereby providing the metabolic reprogramming (lipid synthesis and mitochondrial function maintenance) required for activated HSCs [89]. As demonstrated in AML12 hepatocytes (a murine cell line) and *eIF6*+/− mouse models, *eIF6* deficiency leads to decreased C/EBPβ-LIP protein and subsequent downregulation of lipogenic genes such as *Fasn* and *Acaca* [89]. However, upon eIF6 loss, the mTORC1–eIF4F pathway remains intact and can sustain mitochondrial respiratory chain gene expression via translational regulation of *YY1* (which contains an mTOR-dependent 5′-UTR), resulting in enhanced fatty acid oxidation (FAO) [89]. Collectively, within the hypoxic microenvironment, HIF-1α may form a positive feedback loop with eIF4E and eIF6 in HSCs: HIF-1α promotes HSC activation, while activated HSCs rely on efficient translation to maintain their highly secretory and proliferative myofibroblast phenotype, continuously depositing ECM and exacerbating fibrosis.

Therefore, intervening in the translational control of pro-fibrotic factors—either by directly inhibiting eIF4E or eIF6 themselves, or indirectly by suppressing their function (e.g., through inhibition of upstream signaling molecules such as mTOR)—holds great therapeutic potential for the treatment of liver fibrosis. *eIF4E* antisense oligonucleotides (ASOs) and *eIF6* small-molecule inhibitors (eIFsixty-1/6) have already demonstrated therapeutic efficacy in liver fibrosis models. The former reduces α-SMA and collagen deposition in animal models, while the latter suppresses malignant transformation from fibrosis to HCC [89,90]. In a rat model of bile duct ligation (BDL)-induced liver fibrosis, the anti-fibrotic compound pirfenidone (PFD) has also been shown to improve the fibrotic phenotype, in part through downregulation of eIF6, P311, and TGF-β [91]. Indirect strategies include the use of mTOR inhibitors (e.g., AZD8055) or overexpression of 4EBP1-4A to restore translational repression, thereby simultaneously downregulating HIF-1α and multiple ECM components. Compared with broad-spectrum kinase inhibitors, targeting eIF4E or eIF6 may offer greater selectivity, as these factors preferentially control the translation of pro-fibrotic mRNAs with complex (G+C-rich) 5′-UTRs while having less impact on housekeeping genes—thus potentially suppressing HSC activation with reduced systemic toxicity. Future strategies should focus on developing highly selective translation initiation inhibitors and exploring their sequential or combined use with anti-hypoxic agents (e.g., HIF-1α inhibitors) to synergistically block HSC activation, reverse fibrosis, and promote liver regeneration.

### 5.6. Cell-Targeted Delivery Strategies

However, the strategies above face the common challenge of how to achieve precise delivery of therapeutic agents to HSCs in order to maximize efficacy and minimize systemic side effects. Given the cell type-specific roles of HIF isoforms, a strategy that enables selective delivery of nucleic acid aptamers, miRNAs, or siRNAs to HSCs, hepatocytes, or macrophages could enhance therapeutic outcomes while reducing off-target effects. For example, aptamers that specifically bind to HSCs (such as APT8) can be used as targeted delivery vehicles to precisely transport therapeutic miRNAs (e.g., *miR-23b-5p*) to diseased cells, achieving high efficacy with low side effects.

To improve the in vivo stability and targeting efficiency of siRNA, novel delivery systems are being developed. These systems include conjugating the drug to molecules that bind to receptors enriched on the surface of activated HSCs (e.g., PDGFRβ), or encapsulating the drug in vitamin A-rich nanoparticles that are preferentially taken up by HSCs [92,93,94]. One example is the use of vitamin A-polyethylene glycol (VA-PEG)-modified carbon nitride-based nanosheets (VA-PEG-CN@GQDs) to encapsulate HIF-1α siRNA. VA (vitamin A) enables active targeting of HSCs by specifically binding to retinol-binding protein (RBP) receptors, which are overexpressed on the surface of these cells, thereby promoting the accumulation of the nanocomplex at fibrotic sites [95]. This targeted delivery strategy has demonstrated stronger anti-fibrotic effects than free siRNA in preclinical studies, while also significantly reducing hepatorenal toxicity.

### 5.7. Modulating the Hypoxic Microenvironment

From a broader perspective, the root cause of sustained HIF-α activation in fibrotic liver is the hypoxic microenvironment. Therefore, strategies that improve hepatic oxygen supply–demand balance and eliminate hypoxic stimuli at the source represent a more fundamental therapeutic approach. Current approaches include the use of vasodilators to improve sinusoidal perfusion, metabolic modulators to reduce oxygen consumption, hyperbaric oxygen therapy (HBOT), and oxygen carriers (e.g., perfluorocarbons, hemoglobin-based oxygen carriers) [96]. HBOT increases the oxygen content dissolved in plasma, thereby delivering oxygen more efficiently to hypoxic tissues. The oxygen carriers physically or chemically carry oxygen to hypoxic sites. In the context of liver fibrosis, these strategies can effectively alleviate the hypoxic microenvironment, thereby inhibiting HIF stabilization and downstream pro-fibrotic signaling. By optimizing hepatic microcirculation and cellular metabolism, interventions targeting the fibrotic microenvironment can fundamentally suppress the activation signals of HIFs, thereby blocking fibrotic progression.

#### 5.7.1. Improvement of Sinusoidal Perfusion by Vasodilators

Sinusoidal microcirculatory disturbance is a major contributor to the formation of a hypoxic microenvironment during liver fibrosis. In the fibrotic liver, dysfunction of LSECs and the release of vasoconstrictors such as endothelin-1 increase sinusoidal microcirculatory resistance, creating localized hypoxic areas. This forms a vicious cycle in which hypoxia worsens LSEC dysfunction, which in turn increases sinusoidal resistance and leads to more severe hypoxia. Therefore, the use of vasodilators to improve sinusoidal perfusion and increase tissue oxygen supply may effectively break this cycle, thereby blocking the upstream hypoxic drive of HSC activation.

Statins can increase endothelial nitric oxide synthase (eNOS)-dependent nitric oxide (NO) release and inhibit the endothelin-1 (ET-1) signaling pathway, thereby improving LSEC function and reversing capillarization. These effects reduce intrahepatic vascular resistance, increase sinusoidal perfusion, and alleviate liver fibrosis [97,98]. Studies have shown that in a rat model of early NASH, treatment with simvastatin or atorvastatin for two weeks restored the differentiated phenotype of LSECs and reversed HSC activation, thereby lowering portal pressure and improving NASH histological features [98]. Furthermore, a biomimetic microbubble co-loaded with simvastatin and platelet membranes was recently developed. Using ultrasound-targeted microbubble destruction (UTMD) technology, this system achieves efficient drug release within the hepatic sinusoids. The multifunctional liposomal microbubble loaded with simvastatin and coated with platelet membranes (Sim-MBPLT) treats liver fibrosis through a dual mechanism: reversing LSEC capillarization and inhibiting platelet-mediated HSC activation [97].

#### 5.7.2. Oxygen Therapy and Oxygen Carrier Supplementation

The application of hyperbaric oxygen therapy in liver diseases has a certain research basis. Preclinical studies have shown that HBOT provides protection to injured liver tissue through antioxidant and anti-inflammatory effects [96]. In a rat model of cholestatic liver injury induced by BDL, HBO treatment alleviated BDL-induced oxidative injury, hepatocyte damage, bile duct proliferation, and fibrosis. The hepatoprotective effect was attributed to its antioxidant potential [96]. A study showed that in a mouse model fed a high-fat, high-cholesterol diet, hyperbaric oxygen therapy significantly alleviated liver dysfunction, hepatic steatosis, inflammation, and fibrosis [99].

However, supplemental oxygen therapy merely serves as a direct means to increase blood oxygen partial pressure. Its application in liver diseases is limited by the diffusion capacity of oxygen in tissues. Moreover, simply ameliorating hypoxia without blocking downstream HIF signaling may not fully suppress the already initiated fibrotic cascade. Therefore, improving the hypoxic microenvironment is more suitable as part of a combination therapy, working synergistically with other strategies that target the HIF pathway or its downstream effector molecules.

#### 5.7.3. Potential of Combination Strategies

Taken together, the above analysis demonstrates that HIF-driven HSC activation involves a multi-level regulatory network ranging from upstream integration of signals such as TNF receptor-associated factor 2 (TRAF2) and mTORC1, to core transcriptional regulation by HIF-1α and HIF-2α, and downstream metabolic reprogramming of glucose and glutamine. This complexity makes it difficult to achieve ideal anti-fibrotic effects through single-target intervention strategies. Several reasons account for this. First, HIF-1α and HIF-2α have distinct or even antagonistic functions in liver fibrosis; inhibiting one isoform alone may lead to compensatory activation of the other. Second, upstream regulators of HIF-α, such as TRAF2, mTORC1, and PHD1, exhibit signaling crosstalk, so blocking a single node may not fully suppress HIF-α activation. Third, even when HIF-α is effectively inhibited, activated HSCs may maintain their fibrotic phenotype through HIF-independent pathways. Therefore, combination therapy strategies, such as pairing upstream regulators with downstream metabolic targets, dual inhibition of both HIF-1α and HIF-2α, synergistic application of gene silencing with microenvironment improvement, or a “source-saving and supply-enhancing” approach combining vasodilators and metabolic modulators, represent promising strategies to overcome the limitations of monotherapy and achieve more effective and durable anti-fibrotic outcomes.

A study has demonstrated that VA-PEG-modified carbon nitride (CN)-based nanosheets encapsulating HIF-1α siRNA can both generate oxygen upon near-infrared (NIR) irradiation by triggering water splitting and, under the action of liver lipases, achieve reversible release of the siRNA. The oxygen generation ameliorates the hypoxic environment in fibrotic liver tissue, while the released siRNA can silence HIF-1α. This system inhibits HSC activation through the HIF-1α-mediated TGF-β1/Smad pathway [96]. This “self-oxygenating” nano-delivery system represents an innovative integration of oxygen supply strategy and gene therapy for liver fibrosis. In vitro and in vivo experiments demonstrated that this oxygen–gene combination therapy maximally downregulates HIF-1α expression and enhances therapeutic efficacy. As a result, it reduces collagen deposition and effectively ameliorates liver fibrosis [95].

**Figure 3:**
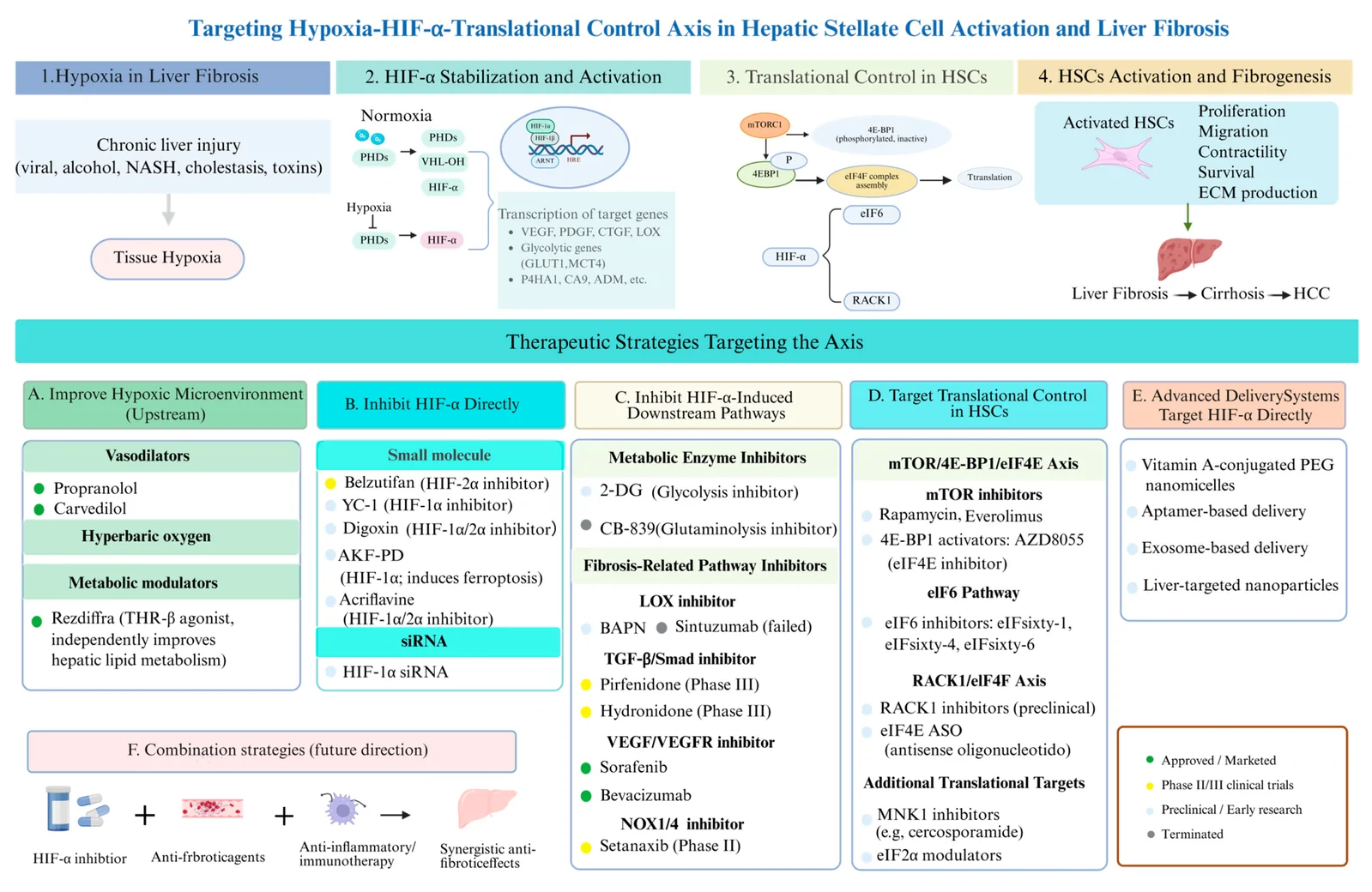
Therapeutic strategies targeting the hypoxia–HIFα–translational control axis in hepatic stellate cell activation and liver fibrosis. Building upon the mechanistic framework established in Figure 1 and Figure 2—spanning hypoxia-driven HIF-α stabilization, transcriptional reprogramming, and translational control via the RACK1–mTORC1–eIF4E and eIF6 axes—this figure categorizes potential therapeutic interventions according to their site of action along the pathogenic cascade. Strategies range from improving the hypoxic microenvironment, directly inhibiting HIF-α or its downstream effectors, to targeting the translational machinery in HSCs, with advanced delivery systems under development to enhance cell-type specificity. Based on this mechanistic framework, the figure outlines multi-layer therapeutic strategies. Upstream interventions aim to alleviate the hypoxic microenvironment using vasodilators (propranolol, carvedilol), hyperbaric oxygen, or metabolic modulators such as resmetirom. Direct HIF-α inhibitors (belzutifan, digoxin, YC-1, acriflavine, and HIF-1α siRNA) block the transcription factor at its source. Downstream blockade targets HIF-induced effectors, including glycolytic enzymes (2-DG, FX-11), and glutaminolysis (CB-839, a GLS1 inhibitor), LOX (BAPN, simtuzumab), the TGF-β/Smad pathway (pirfenidone, hydronidone), VEGF/VEGFR (sorafenib, bevacizumab), and NOX1/4 (setanaxib). Translational control-directed approaches include mTOR inhibitors (rapamycin, everolimus), 4E-BP1/eIF4E modulators (AZD8055), eIF6 inhibitors (eIFsixty-1/4/6), and antisense oligonucleotides targeting eIF4E. Advanced delivery systems such as vitamin A-conjugated PEG nanomicelles, aptamer- or exosome-based carriers, and liver-targeted nanoparticles are being developed to achieve HSC-specific delivery of HIF-α-targeting agents. Future directions include identifying predictive biomarkers (e.g., HIF-1α expression, hypoxia signatures) for patient stratification, developing next-generation HIF modulators with improved selectivity, and evaluating their long-term safety and efficacy in clinical trials. Arrow definitions: Solid arrows indicate established activation or progression pathways; T-bars denote inhibitory interventions. Detailed abbreviations are defined in the main text and abbreviation list. Created in BioRender. LI, W. (2026). (For computer program and AI disclosure, see the statement preceding the figures.)

Additionally, a multifunctional liposomal microbubble loaded with simvastatin and coated with Sim-MBPLT has been developed for liver fibrosis combination therapy using UTMD technology [97]. In a rat model of liver fibrosis, this microbubble system acts through two mechanisms. Simvastatin reverses LSECs capillarization by upregulating eNOS-dependent NO production. On the other hand, the homologous targeting effect of the platelet membrane enables the microbubbles to adhere specifically to inflammatory phenotype LSECs. This competitively blocks the adhesion and activation of endogenous platelets and reduces the release of platelet-derived growth factor β (PDGF-β), thereby inhibiting HSC activation [97]. Experimental results showed that Sim-MBPLT significantly reduced the degree of liver fibrosis, decreased collagen deposition, and improved liver function, with good targeting ability and biosafety. A comprehensive overview of the therapeutic strategies discussed in this review is presented in Figure 3.

## 6. Challenges and Future Directions in Clinical Translation

In summary, the regulatory network by which HIFs drive HSC activation exhibits multi-level, multi-node, and multi-cell-type complexity. This fundamentally limits the efficacy of single-target intervention strategies.

Several key challenges currently constrain clinical translation. First, HIFs have dual temporal effects, which makes it hard to define a clear therapeutic window. Second, HIF-1α and HIF-2α serve divergent functions across different cell types. Therefore, any effective strategy needs both isoform-specific and cell type-specific selectivity. Third, there is extensive crosstalk between the HIF pathway and other pro-fibrotic signals, such as TGF-β and PDGF. This crosstalk greatly reduces the efficacy of monotherapy. Fourth, we still lack reliable biomarkers that could guide patient stratification or monitor treatment response. Lastly, systemic inhibition of HIFs carries non-negligible on-target toxicity. Therefore, future efforts should focus on developing cell-specific delivery systems, discovering novel HIF-modulating molecules, establishing robust pharmacodynamic biomarkers, and systematically exploring “multi-level, multi-target” combination intervention strategies. Such an approach may represent a key path forward to meet the anti-fibrotic treatment needs of patients with progressive liver disease.

Specifically, upstream regulation blocks multiple HIF-α-mediated pathways at the source and is suited for early-stage fibrosis, whereas downstream targeting offers better pharmacological accessibility and applicability to established fibrosis. An optimal strategy may be a stratified combination: upstream intervention in early stages to prevent HIF-α-driven initiation, plus downstream targeting (e.g., HSC-specific TGF-β1 inhibition) in advanced fibrosis. This represents a direction towards precision therapy for liver fibrosis.

## 7. Conclusions

Hypoxia drives HSC activation and liver fibrosis through HIF-mediated transcriptional responses. A number of pro-fibrotic pathways converge on HIF signaling, which offers both possibilities and hurdles for therapy. We now know quite a bit about how hypoxia triggers HSC activation, and some experimental drugs targeting these pathways have looked promising in animal models.

From this analysis, several research priorities stand out. First, a deeper understanding of how HIF isoforms function in specific cell types will help design more selective therapeutic strategies. Second, improved delivery systems that enable targeted modulation of HIF activity, specifically in HSCs, hepatocytes, or macrophages, could enhance efficacy while reducing systemic toxicity. Third, combination strategies that simultaneously target the HIF pathway and other pro-fibrotic cascades need to be systematically evaluated. Fourth, the development of reliable biomarkers for patient stratification and treatment monitoring will accelerate clinical translation. HIFs serve as central integrators of metabolic, inflammatory, and fibrotic signals, which makes them appealing drug targets. With the ongoing advances in drug delivery and molecular selectivity, HIF-directed therapies could eventually help fill the large gap in anti-fibrotic treatment for patients with progressive liver disease.

## Figures and Tables

**Table 1:** Classification and characteristics of small-molecule HIF inhibitors for the treatment of liver fibrosis.

Inhibitors	The Target	Mechanism of Action	Research Progress on Liver Fibrosis	The Clinical Status
YC-1	HIF-1α	Blocking HIF-1α protein synthesis; Activating sGC	Inhibits LX-2 cell activation and induces apoptosis; alleviates BDL-induced liver fibrosis in mice	Preclinical
AKF-PD	HIF-1α	Inhibition of HIF-1α/SLC7A11 pathway induces ferroptosis	Alleviates CCl_4_-induced liver fibrosis in rats by promoting ferroptosis in hepatic stellate cells (HSCs)	Phase II clinical
Digoxin	HIF-1α/HIF-2α	Inhibition of HIF-1α protein translation; Blockade of the PKM2-HIF-1α axis	Improves HFD-induced NASH; downregulates hepatic fibrotic gene expression	FDA-approved for heart disease
PT2385/PT2977 (Belzutifan)	HIF-2α	Blocking HIF-2α and HIF-1β dimerization	Approved for VHL-associated renal cell carcinoma; its application in liver fibrosis remains theoretical	FDA-approved for cancer
Acriflavine	HIF-1α/HIF-2α	Blocking HIF interaction with p300/CBP	Broad-spectrum HIF inhibitor; anti-fibrotic mechanism requires validation	Preclinical
Caspofungin	HIF-1α (indirect)	Potential HIF-1 pathway regulation (mechanisms to be elucidated)	Associated with HIF-1 activation in liver injury; anti-fibrotic effect remains to be validated	FDA-approved for antifungal therapy

## Data Availability

Reasonable requests to access the related data will be considered on a case-by-case basis and should be made to the corresponding author (Jingwei Mao, maojingwei@dmu.edu.cn).

## Abbreviations

The following abbreviations are used in this manuscript:

4-EBP1	Eukaryotic translation initiation factor 4E-binding protein 1
AKF-PD	Fluorofenidone
ARNT	Aryl hydrocarbon receptor nuclear translocator
BAPN	B-aminopropionitrile
BDL	Bile duct ligation
CCl_4_	Carbon tetrachloride
CPEB4	Cytoplasmic polyadenylation element binding protein 4
CPH	Cirrhotic portal hypertension
CXCR4	C-X-C chemokine receptor type 4
DCA	Dichloroacetate
eIF4E	Eukaryotic translation initiation factor 4E
eIF6	Eukaryotic initiation factor 6
ECM	Extracellular matrix
EMT	Epithelial–mesenchymal transition
eNOS	Endothelial nitric oxide synthase
ERK	Extracellular signal-regulated kinase
GATA4	GATA binding protein 4
GLS1	Glutaminase 1
GLUT1	Glucose transporter type 1
HBOT	Hyperbaric oxygen therapy
HCC	Hepatocellular carcinoma
HIF	Hypoxia-inducible factor
HK2	Hexokinase 2
HRE	Hypoxia response element
HSC	Hepatic stellate cell
IKK	IκB kinase
IKKα/β	KKα/β-IκB kinase α and β
IκBα	Inhibitor of kappa B alpha
LDHA	Lactate dehydrogenase A
LOX	Lysyl oxidase
LSEC	Liver sinusoidal endothelial cell
MAPK	Mitogen-activated protein kinase
MASP1	Mannan-binding lectin-associated serine protease 1
miRNA	microRNA
mTORC1	Mechanistic target of rapamycin complex 1
NASH	Nonalcoholic steatohepatitis
NF-κB	Nuclear factor kappa-B
NOX	NADPH oxidase
p38	Mitogen-activated protein kinase
PDK1	Pyruvate dehydrogenase kinase 1
PFKFB3	6-phosphofructo-2-kinase/fructose-2,6-bisphosphatase 3
PHD	Prolyl hydroxylase domain-containing protein
PKM2	Pyruvate kinase M2
RACK1	Receptor for activated C kinase 1
ROS	Reactive oxygen species
sEVs	Small extracellular vesicles
sGC	Soluble guanylyl cyclase
siRNA	Small interfering RNA
SLC7A11	Solute carrier family 7 member 11
Smad	Sma- and Mad-related protein
TCA	Tricarboxylic acid cycle
TGF-β1	Transforming growth factor-β1
TRAF2	TNF receptor-associated factor 2
UTMD	Ultrasound targeted microbubble destruction
VA-PEG	vitamin A-polyethylene glycol
VEGF	Vascular endothelial growth factor
VEGFR	Vascular endothelial growth factor receptor
VHL	von Hippel–Lindau
YAP	Yes-associated protein
YC-1	Lificiguat
α-SMA	Alpha-smooth muscle actin
